# Thoracic Rat Spinal Cord Contusion Injury Induces Remote Spinal Gliogenesis but Not Neurogenesis or Gliogenesis in the Brain

**DOI:** 10.1371/journal.pone.0102896

**Published:** 2014-07-22

**Authors:** Steffen Franz, Mareva Ciatipis, Kathrin Pfeifer, Birthe Kierdorf, Beatrice Sandner, Ulrich Bogdahn, Armin Blesch, Beate Winner, Norbert Weidner

**Affiliations:** 1 Spinal Cord Injury Center, Heidelberg University Hospital, Heidelberg, Germany; 2 Department of Neurology, University of Regensburg, Regensburg, Germany; 3 IZKF Junior Group III and BMBF Research Group Neuroscience, Interdisciplinary Center for Clinical Research, Nikolaus-Fiebiger Center for Molecular Medicine, Friedrich-Alexander University-Erlangen-Nürnberg, Erlangen, Germany; Hertie Institute for Clinical Brain Research, University of Tuebingen, Germany

## Abstract

After spinal cord injury, transected axons fail to regenerate, yet significant, spontaneous functional improvement can be observed over time. Distinct central nervous system regions retain the capacity to generate new neurons and glia from an endogenous pool of progenitor cells and to compensate neural cell loss following certain lesions. The aim of the present study was to investigate whether endogenous cell replacement (neurogenesis or gliogenesis) in the brain (subventricular zone, SVZ; corpus callosum, CC; hippocampus, HC; and motor cortex, MC) or cervical spinal cord might represent a structural correlate for spontaneous locomotor recovery after a thoracic spinal cord injury. Adult Fischer 344 rats received severe contusion injuries (200 kDyn) of the mid-thoracic spinal cord using an Infinite Horizon Impactor. Uninjured rats served as controls. From 4 to 14 days post-injury, both groups received injections of bromodeoxyuridine (BrdU) to label dividing cells. Over the course of six weeks post-injury, spontaneous recovery of locomotor function occurred. Survival of newly generated cells was unaltered in the SVZ, HC, CC, and the MC. Neurogenesis, as determined by identification and quantification of doublecortin immunoreactive neuroblasts or BrdU/neuronal nuclear antigen double positive newly generated neurons, was not present in non-neurogenic regions (MC, CC, and cervical spinal cord) and unaltered in neurogenic regions (dentate gyrus and SVZ) of the brain. The lack of neuronal replacement in the brain and spinal cord after spinal cord injury precludes any relevance for spontaneous recovery of locomotor function. Gliogenesis was increased in the cervical spinal cord remote from the injury site, however, is unlikely to contribute to functional improvement.

## Introduction

Spinal cord injury (SCI) with complete axonal transection precludes any degree of sensorimotor or autonomous functional recovery [1], [2]. In contrast, neurological and consecutively functional recovery is consistently observed in individuals with incomplete SCI, reflected by conversion in the American Spinal Injury Association Impairment Scale and by gains in respective functional assessments, such as the Spinal Cord Independence Measure and the Walking Index for Spinal Cord Injury [3]–[5]. The observed recovery should not be called “spontaneous”, since patients with SCI undergo intense rehabilitation programs intended to promote functional recovery.

The exact structural correlates contributing to functional improvement in incomplete SCI have yet to be determined. It has been shown that axonal sprouting and synaptic rearrangements above and below the injury site in rodent and primate models contribute to recovery of function [6]–[10]. Whether intrinsic neural cell replacement remote from the injury site underlies the recovery process, at least in part, is yet unknown.

Adult neurogenesis - the generation of new neurons in the adult brain - represents a continuously occurring process in the dentate gyrus of the hippocampus (HC) and in the subventricular zone (SVZ), the latter of which demonstrates subsequent migration and neuronal differentiation in the olfactory bulb in animals and humans [11]–[13]. Several studies have documented the generation [14], [15] and integration of newborn neurons into complex neuronal networks in neurogenic regions of the rodent and primate central nervous system (CNS) [16]–[18]. Neurons undergoing apoptotic cell death in the motor cortex (MC) following a highly selective cortical lesion appear to be replaced by newborn neurons, some with the capacity to extend axons as far as the cervical spinal cord in adult mice [17]. If remote neuronal cell death is a significant consequence of SCI, as previously indicated [19], neuronal replacement, as described in a proof of concept study [17], could contribute to spontaneous recovery observed in incomplete SCI.

In the injured adult spinal cord, neuronal and glial cell death occurs in the vicinity of and remote from the lesion site [20]. However, in the intact or injured adult spinal cord, cell replacement has only been described for glial cells, whereas neurogenesis has not been convincingly demonstrated and seems unlikely [21]–[29]. For spontaneous recovery after SCI, glial replacement might play a role in the context of oligodendroglial turnover and remyelination of axons, which survive to some degree even in clinically complete SCI subjects [30]. In spinal cord regions remote from the injury site, the relevance of glial cell replacement for spontaneous functional recovery has yet to be demonstrated.

In the present study, we hypothesized that spontaneous functional recovery observed in spinal cord contused adult rats is linked to relevant neuronal or glial cell replacement in cortical, subcortical, or spinal regions. To address this question, adult female rats underwent thoracic spinal cord contusions followed by intraperitoneal bromodeoxyuridine (BrdU) injections in order to visualize newborn cells. Animals were assessed in terms of locomotor function over 6 weeks after SCI. Thereafter, newly generated cells and their differentiation patterns were assessed in the MC, SVZ, corpus callosum (CC), HC, and cervical spinal cord.

## Materials and Methods

### Ethics Statement

Experiments were carried out in accordance with the European Communities Council Directive (86/609/EEC) and institutional guidelines. The animal protocol was approved by the District Government of Upper Palatinate (Permit Number: 54-2531.1-33/05). All surgical procedures were performed using a cocktail of ketamine, xylazine, and acepromazine, as defined below. All efforts were made to minimize discomfort.

### Animal Subjects and Experimental Groups

Twenty female adult Fischer 344 rats (Charles River Germany GmbH, Sulzfeld, Germany) weighing between 160 and 180 g were used. All animals were housed in groups of 5 on a 12-h light/dark cycle with access to food and water *ad libitum*. For experimental purposes, the animals were separated into two groups (injured and control group), 10 animals each. All animals of the injured group received a spinal contusion, as described below. Animals of the control group did not undergo any surgical intervention.

### Surgical Procedures and BrdU injections

Rats were deeply anesthetized by intramuscular injection of ketamine (62.5 mg/kg; 100 mg/l; WDT, Garbsen, Germany), xylazine (3.125 mg/kg; 20 mg/ml; Serumwerk Bernburg AG, Germany), and acepromazine (0.625 mg/kg; 13.56 mg/ml; Sanofi-Ceva, Düsseldorf, Germany). Animals received a laminectomy at Th10. Following fixation of the adjacent Th9 and Th11 vertebral body to suspend the target region, a standardized thoracic spinal contusion (200 kDyn) was applied using an Infinite Horizon Impactor (Precision Systems & Instrumentation, Lexington, KY, USA), as described [31], [32]. Following the injury, muscle layers were sutured and the skin was closed. Post-interventional care included manual voiding of the bladder twice a day for the first 10 days, subcutaneous injections of cotrimoxazole (10 mg/kg; Ratiopharm, Ulm, Germany) to avoid bladder infections, and administration of analgesics (buprenorphine; 0.03 mg/kg max. twice a day) as needed. From day 4 to day 14, animals of both experimental groups received daily intraperitoneal injections of BrdU (50 mg/kg KG; 10 mg/ml in 0.9% saline). The prolonged period of BrdU injections was chosen to follow the fate of dividing cells in non-neurogenic regions, where a very limited number of cells undergoing neuronal differentiation was expected.

### Functional Testing

Locomotor function was assessed by two observers independently using the modified 12-point Basso, Beattie & Bresnahan (BBB) open field locomotor scale on post-op days 1, 8, 15, 22, 29, 36, and 40 [33], [34].

### Tissue Processing

Six weeks post-operatively, rats were deeply anesthetized as described above and transcardially perfused with 0.9% saline solution, followed by 4% paraformaldehyde in 100 mM phosphate buffered saline (PBS). Brains and spinal cords were dissected, post-fixed with 4% paraformaldehyde overnight, and subsequently submersed in 30% sucrose at 4°C for at least 24 h. Brains were cut into 40 µm coronal sections on a sliding microtome (Leica, Germany), with every 12th section (480 µm intervals) processed for immunolabeling. A 5 mm block of spinal cord was excised at cervical level C4 and cut into 35 µm coronal sections with a cryostat (Leica, Germany). Every 10th section (350 µm intervals) was processed for immunolabeling. Brain and spinal cord sections were stored at −20°C in cryoprotectant solution (25% glycerol, 25% ethylene glycol, and 0.1 M phosphate buffer, pH 7.4).

### Immunolabeling

For immunolabeling, free-floating brain and spinal cord sections were treated with 0.6% H_2_O_2_ in Tris-buffered saline (TBS: 0.15 M NaCl, 0.1 M Tris–HCl, pH 7.5) for 30 min. After rinsing in TBS, free-floating sections were incubated in 0.6% H_2_O_2_ for 30 min, washed again, and incubated for 1 h in 50% formamide/2xSSC (0.3 M NaCl, 0.03 M sodium citrate) at 65°C. Sections were rinsed in 2x SSC, incubated for 30 min in 2 M HCl at 37°C, and rinsed for 10 min in 0.1 M boric acid (pH 8.5). Following extensive washes in TBS, sections were blocked with a solution composed of TBS, 0.2% fish skin gelatin (Sigma-Aldrich, Germany), 1% bovine serum albumin (Biomol, Hamburg, Germany), and 0.1% Triton X-100 (Sigma-Aldrich, Germany) for 1 h followed by overnight incubation with the primary antibody at 4°C. On the following day, sections were washed extensively and incubated with biotin-conjugated species-specific secondary antibodies (Dianova, Hamburg, Germany and Vector Laboratories, Burlingame, USA) for 1 h, followed by incubation with a peroxidase–avidin complex solution (Vectastain Elite ABC kit; Vector Laboratories, Burlingame, USA). The peroxidase activity of immune complexes was visualized with a solution of TBS containing 0.25 mg/ml 3, 3′-diaminobenzidine (Vector Laboratories, Burlingame, USA), 0.01% H_2_O_2_, and 0.04% NiCl_2_. Sections were mounted on gelatin coated slides and coverslipped in Neo-Mount (Merck, Darmstadt, Germany).

For the analysis of neuronal and glial differentiation, immunofluorescence labeling techniques were performed. For immunofluorescence labeling including BrdU antibodies, the DNA denaturation steps required for BrdU immunohistochemical detection were performed as described above. A combination of rat anti-BrdU and appropriate neuron- or glia-specific antibodies were applied in TBS-donkey serum for 24 h at 4°C. Fluorophor-labeled secondary antibodies, as defined below. After several washes in TBS, sections were mounted on slides and coverslipped with ProLong®Antifade (Molecular Probes).

The following primary antibodies were used: rat anti-BrdU antibody (1/500; Harlan SeraLab, Loughborough, UK), goat anti-doublecortin (DCX; 1/1000, Santa Cruz Biotechnology, Dallas, TX, USA), mouse anti-NeuN (1/500, Chemicon, Temecula, CA), rabbit anti-glial fibrillary acidic protein (GFAP) (1/1000, Dako, Hamburg, Germany), and mouse anti-adenomatous-polyposis-coli (APC)-protein (1/500; Calbiochem, Darmstadt, Germany), rabbit anti-NG2 (1∶200; Chemicon, Temecula, CA, USA), and rabbit anti-ionized calcium binding adapter molecule 1 (IBA1) (1∶500; Wako, Richmond, VA, USA). Immunoreactivity was visualized using Rhodamin-conjugated anti-rat (1/500; Dianova, Hamburg, Germany), Alexa 488–conjugated anti-goat (1/1000; Molecular Probes, Karlsruhe, Germany), Cy5-conjugated anti-rabbit (1/500; Dianova, Hamburg, Germany), and Alexa 488–conjugated anti-mouse (1/1000; Molecular Probes, Karlsruhe, Germany). All Alexa secondary antibodies were of donkey origin.

Early postmitotic neurons were identified by DCX immunoreactivity. Newly generated mature neurons were visualized by co-localization of BrdU and NeuN, astroglial cells by co-localization of BrdU with GFAP, oligodendrocytes by co-labeling of BrdU with APC in the absence of GFAP labeling, glial progenitor cells by co-labeling of BrdU with NG2 and activated microglia by co-localization of BrdU with IBA1 [35]–[40].

### Counting Procedures

For quantification of the brightfield BrdU and DCX immunohistochemistry, a systematic unified random counting procedure, similar to the optical dissector [41], was used as described previously [42]. Every 12th brain section and every 10th spinal cord section were selected from each animal and analyzed for BrdU or DCX positive cells in the MC (7 sections per animal), SVZ (3 sections per animal), CC (5 sections per animal), HC (6 sections per animal) and cervical spinal cord (14 sections per animal).

The reference volume was determined by tracing the areas using a semi-automatic stereology system (Stereoinvestigator, MicroBrightField, Colchester, VT, USA) using a light microscope (Leica, Wetzlar, Germany). Newly generated cells (BrdU positive) and neurons (DCX positive) were counted exhaustively on each section in the SVZ, CC, HC, and MC. Within the HC only the dentate gyrus was included in the analysis. For analysis of the CC and SVZ the total extent according to the respective anatomical borders was chosen. The MC was defined as follows [43]: anterior-posterior from +3,7 to +1,6 mm, dorso-ventral was defined by a horizontal line drawn between the most dorsal edge of the cortex in each hemisphere (dorsal border) and another horizontal line drawn through the genu corpus callosum (ventral border). Within the spinal cord, BrdU positive cells were separately quantified in the region of the central canal, as well as in the surrounding parenchyma. The sums of BrdU and DCX positive cells of each section within the different brain and spinal cord regions were multiplied by a factor of 12 and 10, respectively, and are presented as absolute numbers.

Analysis of immunofluorescently labeled sections was performed using laser confocal microscopy (Leica TCS-NT). Co-localization of BrdU positive cells with respective differentiation markers in the spinal cord was determined by analyzing specimens of 5 animals with the highest number of BrdU positive cells in each group. 40 BrdU positive cells per animal – 20 cells in the ventral and 20 cells in the dorsal part of a horizontal spinal cord section (divided by a line through the central canal) – were randomly analyzed. Adjacent to the central canal, 40 BrdU positive cells were selected and also immunolabeled with differentiation markers. Co-localization for GFAP, APC, NG2 and IBA1 was confirmed, once the differentiation marker was spatially associated with BrdU nuclear labeling through subsequent optical sections in the z-axis. Co-localized cells are presented as percentage of BrdU positive cells. Total numbers were then calculated by multiplying the amount of BrdU positive cells with the respective ratios.

### Statistical Analysis

All data are presented as mean values ± standard error of the mean (SEM). For intra-individual comparison of BBB scores in injured animals at two particular times of observation, one-way repeated measures ANOVA including Bonferroni post-hoc-test were applied. For all further inter-individual comparisons between injured and control animals, an unpaired t-test was used. A value of p<0.05 was considered significant (p<0.05 = *; p<0.01 = **; p<0.001 = ***). For statistical analysis, Prism software (Prism GraphPad Software, USA) was used.

## Results

### Spontaneous recovery of locomotor function after contusion spinal cord injury in adult rats

**Figure 1:**
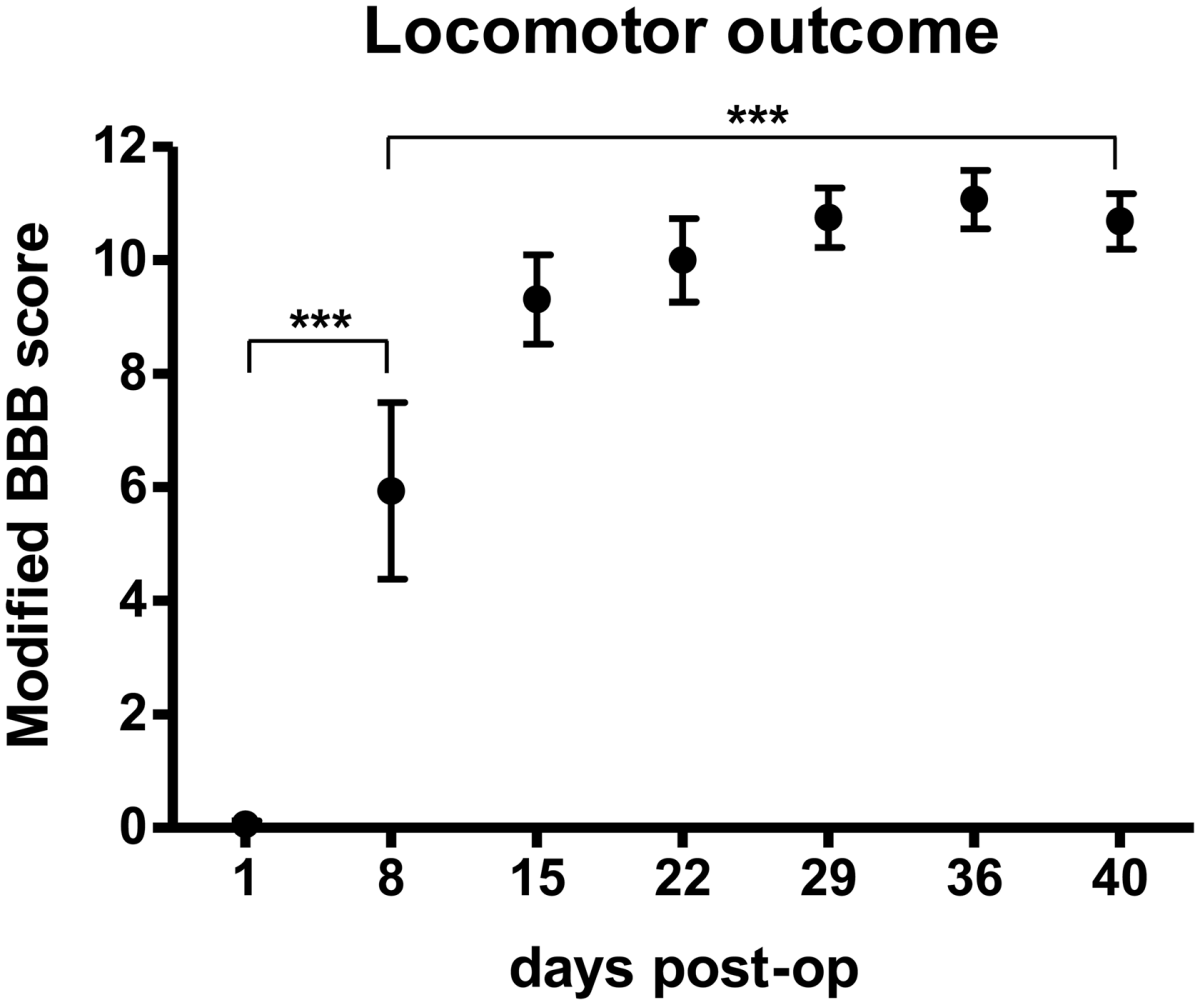
Spontaneous recovery of locomotion. Postoperative assessment of the modified 12-point BBB open field locomotor rating scale in 8 adult rats after a 200 kDyn mid-thoracic spinal cord contusion. Animals were monitored weekly starting day 1 post-injury until post-op day 40.

A standardized thoracic spinal contusion (200 kDyn) at T10 level induced complete paresis of both hindlimbs in all animals one day post-injury (Fig. 1). Two animals died within 24 h after surgery. Prior to surgery, none of the animals showed noticeable gait impairment. At post-op day 8, locomotor function substantially improved to an average BBB score of 5.9±1.6, (p<0.0001) compared to day 1 (Fig. 1). Animals displayed coordinated hindlimb movement with partial body weight support. Compared to BBB scores at day 8, locomotor function further improved at day 40, showing spontaneous functional recovery with nearly unimpaired walking patterns (BBB score 11.1±0.5; p<0.0001; Fig. 1).

### Cell proliferation and neurogenesis

**Figure 2:**
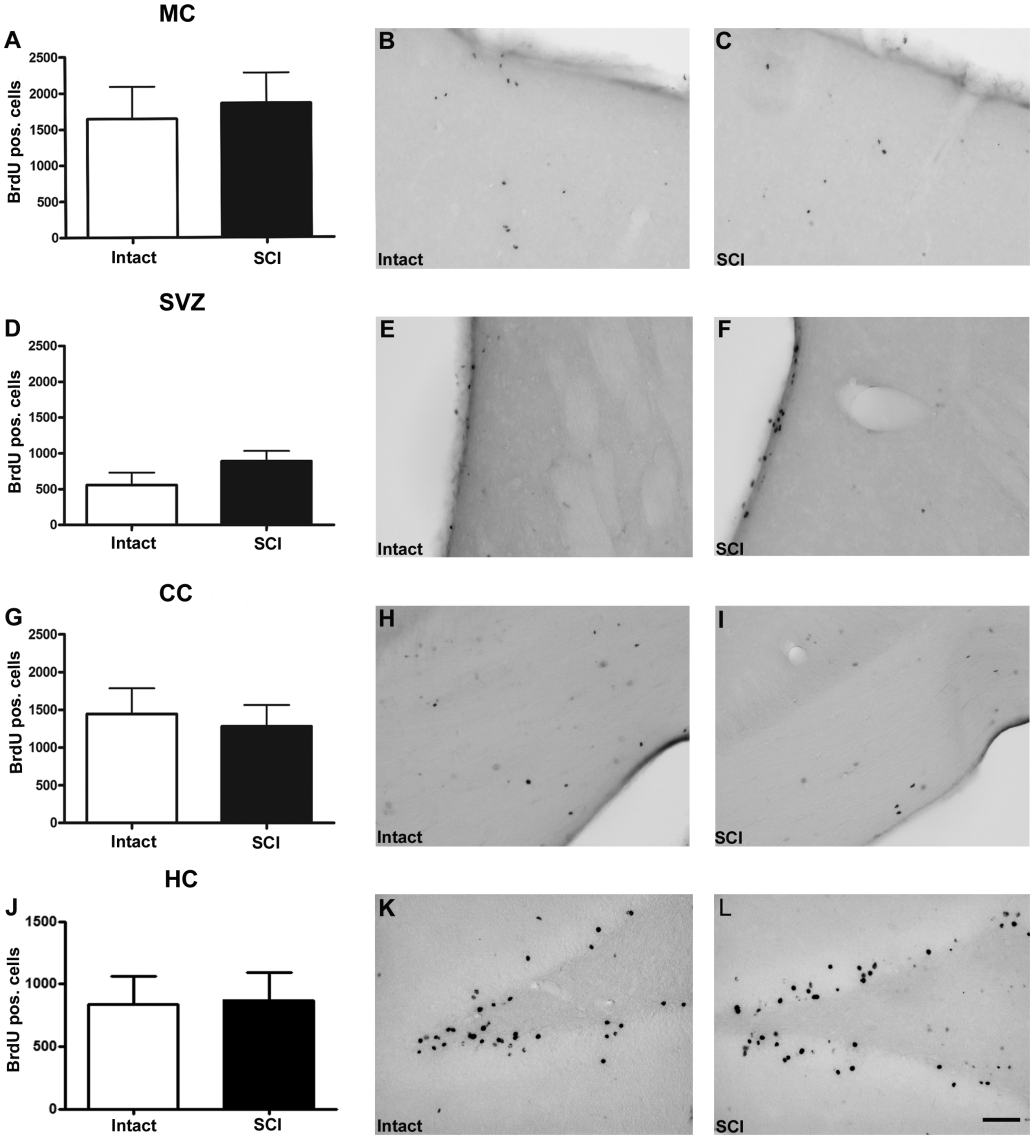
Post SCI cell renewal in motor cortex, subventricular zone, corpus callosum and hippocampus. (A, D, G, J) Quantification of BrdU positive cells in control (Intact) versus spinal cord injured (SCI) animals in the motor cortex (MC; A), subventricular zone (SVZ; D), corpus callosum (CC; G) and hippocampus (HC; H). Brightfield micrographs display cell renewal represented by BrdU immunoreactive nuclei in the MC (B, C), SVZ (E, F), CC (H, I) and HC (K, L) of control (Intact; B, E, H, K) and injured animals (C, F, I, L). Scale bar: 50 µm in (L).

Effects of thoracic SCI on cell renewal in the brain (MC, SVZ, CC, HC) and cervical spinal cord of adult rats were assessed by counting BrdU positive cells 42 days post-injury (animals received BrdU injections between post-op day 4 and 14). No difference was observed in the number of newly generated cells between intact and injured animals in any of the brain regions investigated (MC, SVZ, CC, HC; Fig. 2).

**Figure 3:**
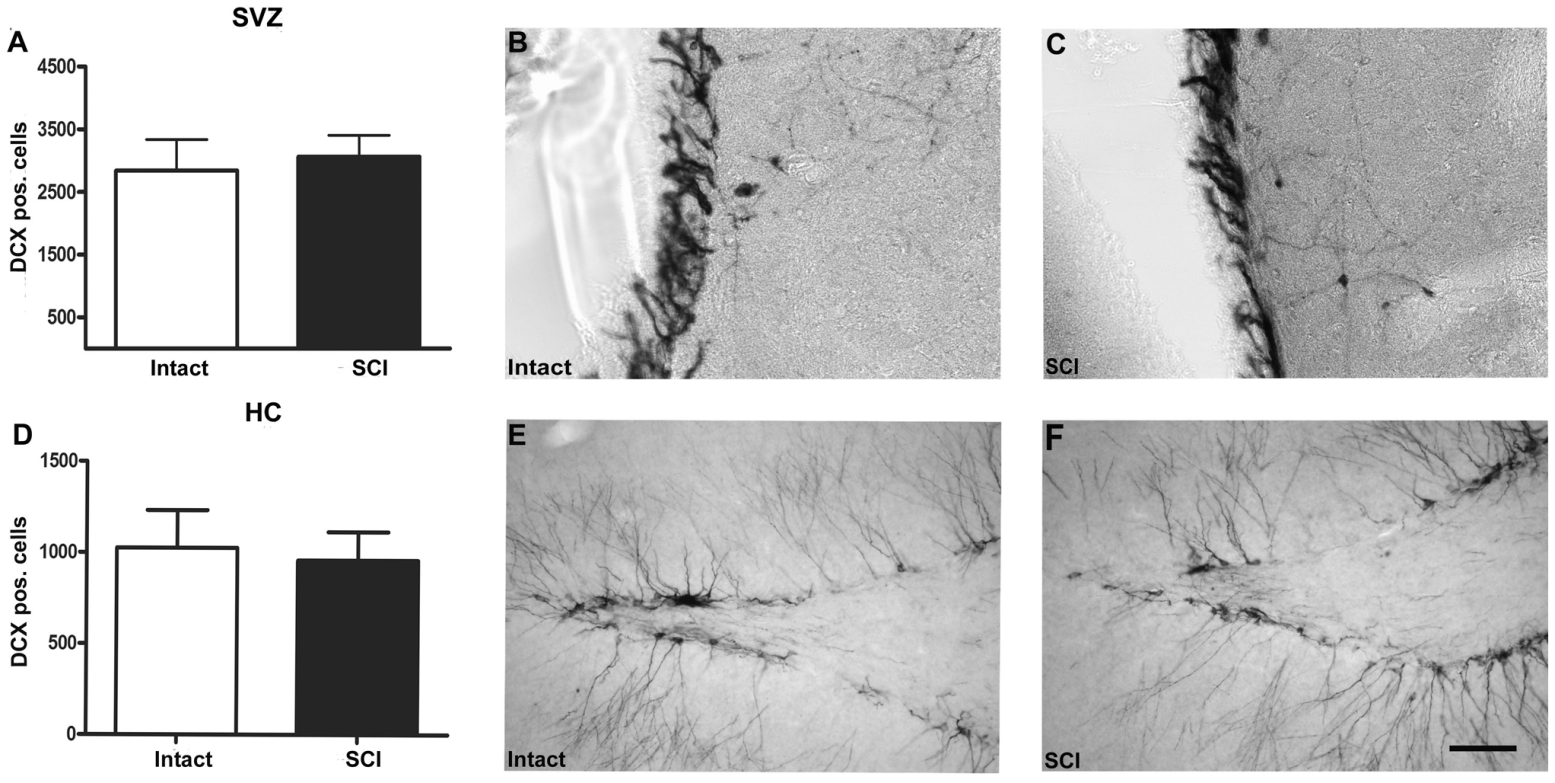
Neurogenesis in the subventricular zone and hippocampus. (A, D) Quantification of DCX immunoreactive cells in the subventricular zone (SVZ; A) and hippocampus (HC; D) in control (Intact) versus spinal cord injured (SCI) animals. Brightfield micrographs display representative section of DCX-expressing neuroblasts in the SVZ (B, C) and HC (E, F) of control (B, E) and spinal cord injured animals (C, F). Scale bar: 100 µm.

DCX immunoreactive cells representing early post-mitotic neurons were only detectable in the SVZ and HC, as expected (Fig. 3). In both regions, quantification did not reveal any difference between intact and injured rats (SVZ: 2842±201 versus 3060±140; HC: 1062±67 versus 1007±58). In the MC, we analyzed on average 290 BrdU positive cells per animal and did not identify any BrdU/NeuN positive cell, indicating that no persisting new neurons were generated in this area following SCI (data not shown).

**Figure 4:**
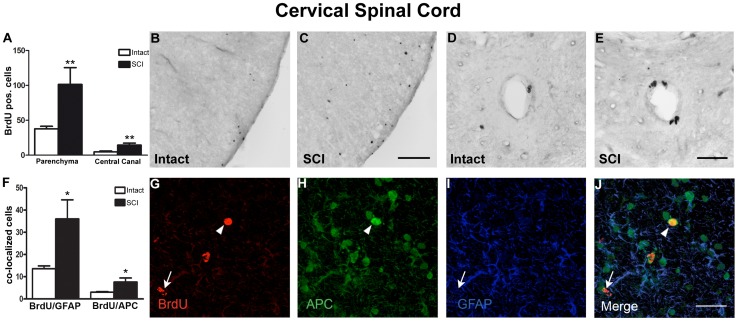
Post SCI cell renewal in the cervical spinal cord. Coronal spinal cord sections were analyzed in the parenchyma and around the central canal. (A) Quantification of BrdU positive surviving newborn cells in the spinal parenchyma and around the central canal of intact and SCI animals. BrdU positive nuclei in the lateral white matter (B, C) and in the ependymal layer of the central canal (D, E) of intact and injured animals (SCI). (F) Quantification of BrdU positive cells expressing GFAP representing astroglia; BrdU positive cells co-localizing with APC, but negative for GFAP, were counted as oligodendrocytes. (G-J) Immunofluorescent labeling for (G) BrdU (red), (H) APC (green) and (I) GFAP (blue) in a coronal cervical spinal cord section of an injured animal taken from the ventral gray matter. (J) Merged image of (G-I). Co-localization of BrdU with GFAP indicates astroglial differentiation (arrow), whereas BrdU/APC positive cells that are GFAP-negative represent oligodendrocytes (arrowhead). Scale bars: 100 µm in (C), 50 µm in (E), 34.1 µm in (J).

**Figure 5:**
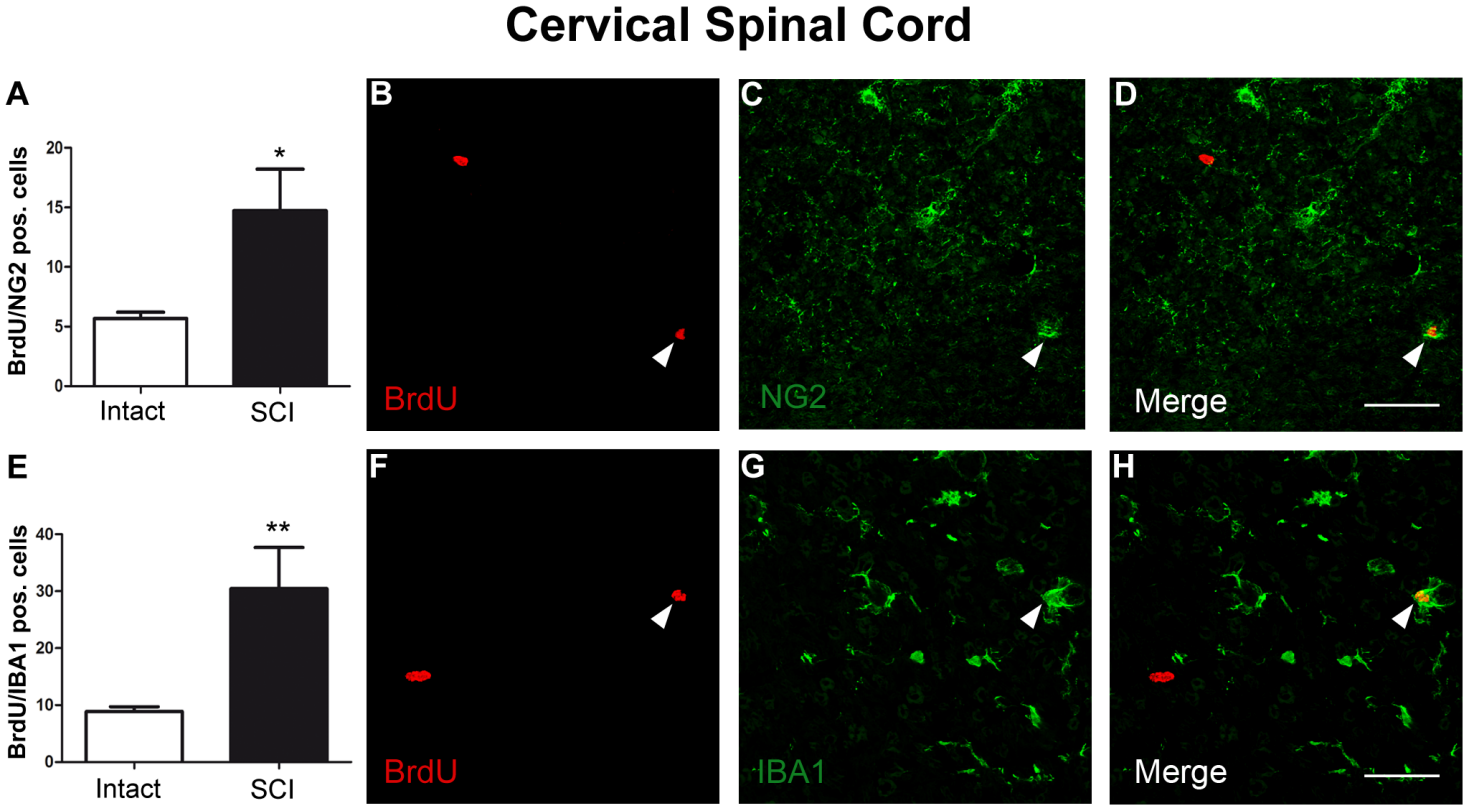
Post SCI glial progenitor cell and microglial renewal in the cervical spinal cord. (A) Quantification of BrdU positive cells expressing NG2 and thus representing glial progenitor cells. (B-D) Immunofluorescent labeling for (B) BrdU (red) and (C) NG2 (green). (D) Merged image of (B, C) indicating a co-localization of BrdU and NG2 (arrowhead). (E) Quantification of BrdU positive cells expressing IBA1 and thus representing newborn microglia. (F-H) Immunofluorescent labeling for (F) BrdU (red) and (G) IBA1 (green). (H) Merged image of (F, G) indicating a co-localization of BrdU and IBA1 (arrowhead). Scale bars: 60 µm in (D) and 45 µm in (H).

In contrast, BrdU positive cells were significantly increased in injured animals compared to intact animals in the cervical spinal cord, both in the spinal parenchyma and in the region of the central canal (101.5±24.1 versus 37.8±3.6, *p<0.05*; central canal: 14,4±2.9 versus 4.7±1.3, *p<0.05*; Fig. 4 A-E). The differentiation pattern within the spinal cord was further analyzed by examining the co-localization of BrdU with the astroglial marker GFAP, the oligodendroglial marker APC (Fig. 4F-J), the glial progenitor cell marker NG2 and the microglial marker IBA1 (Fig. 5). Glial cell renewal was observed only in the spinal parenchyma of the cervical spinal cord. In the ependymal layer around the central canal, BrdU positive nuclei could not be co-localized with any of the tested glial markers (data not shown). The proportion of newborn cells displaying astro-/oligodendroglial, glial progenitor, or microglial differentiation was similar in the cervical spinal cord of injured versus intact animals (BrdU/GFAP co-localized cells: 35.5±3% versus 36±2.2%; BrdU/APC co-localized, GFAP negative cells: 7.5±0.8% vs. 8.0±1.5%; BrdU/NG2 co-localized cells: 14.5±5.1% vs. 15.0±2.4%; BrdU/IBA1 co-localized cells: 30.0±3.8% vs. 23.5±2.3%). Considering the significant difference in the number of BrdU positive cells, the absolute numbers of astroglial, oligodendroglia, glial progenitor cells, and microglia were significantly increased in the cervical spinal cord of injured animals (BrdU/GPAP co-localized cells: 36.0±3.8 vs. 13.6±0.9; *p*<0.05; BrdU/APC co-localized cells: 7.6±1.0 vs. 3.0±0.6, *p*<0.05; BrdU/NG2 co-localized cells: 14.7±3.5 vs. 5.7±0.5, *p<0.05*; BrdU/IBA1 co-localized cells: 30.4±7.2 vs. 8.9±0.8, *p<0.005*; Fig. 4F, Fig. 5A,E). There was no detectable DCX immunoreactivity in the cervical spinal cord, both in intact and injured animals (data not shown).

## Discussion

The present study investigated cell proliferation/neurogenesis in remote CNS areas following a contusion injury that closely reflects human spinal cord pathology. Rats show robust spontaneous recovery of locomotor function, which is not paralleled by neurogenesis in the MC or CC. Moreover, neurogenesis is not altered in SCI rats compared to uninjured animals in the SVZ and HC. Interestingly, increased glial cell renewal can be observed in the cervical spinal cord remote from the thoracic lesion site. The functional relevance of the latter finding has yet to be determined.

The only other published study that analyzed supraspinal neurogenesis in spinal cord injured rats identified persistent depression of neurogenesis in the HC over 90 days after a cervical hemisection injury in adult rats [44]. In contrast, the mid-thoracic contusion SCI in the present study did not alter neurogenesis in the HC. The divergent findings may be explained by the different lesion level (cervical versus thoracic) and/or the different lesion paradigms, which likely effect axon pathways communicating with higher brain regions differentially.

Neuronal renewal should only be relevant for spontaneous functional recovery if the respective disease causes neuronal degeneration and cell death that contributes to functional impairment. For example, cell death represents an indisputable pathophysiological hallmark underlying functional impairment in ischemic stroke [45], [46]. In contrast, neuronal cell death has a limited impact on functional decline in SCI, where the transection of long distance motor, sensory, and autonomous axon pathways defines the clinical phenotype [47], [48]. Whether spinal cord axon transection results in neuronal cell death in the brain remains controversial. After a rat dorsal column transection, substantial degeneration of corticospinal neurons, as determined by the expression of apoptotic markers in layer V of the MC, has been reported within 2 weeks after injury [19]. In contrast, corticospinal axon quantification at the brainstem level following thoracic and cervical rat contusion SCI could not identify a significant decline of axon profiles, suggesting that cell death of corresponding neurons does not occur [49]. A more recent study from the same group employing an identical lesion model and identical assessments to determine pyramidal neuron cell death, as reported by Hains et al, could not replicate the finding of cortical neuronal death following rat SCI [50]. In human SCI subjects, atrophy of the corticospinal tract has been reported by magnetic resonance imaging. However, the limited resolution of MRI does not allow counting of individual axons. The observed atrophy could have been caused by degeneration of glial cells or shrinkage of corticospinal neurons/axons [51], rather than axon depletion due to pyramidal neuron cell death as the morphological substrate [52].

Irrespective of the debate regarding cell death of pyramidal neurons other factors may have contributed to the failure to detect cortical neurogenesis after SCI. The only study that has so far reported replacement of lost cortical neurons in adult mammals following a CNS lesion used a highly specific, limited injury model - apoptotic cell death via chromophore photoactivation. The first newborn mouse neurons projecting axons to the cervical spinal cord were detected at 12 weeks, peaking at the latest investigated time point – 56 weeks post cortical lesion. Overall, the number of detected newborn neurons was very small, between 1–6 neurons per mm^3^ [17]. In the present study, the maximum time between neural progenitor generation and analysis of neuronal fate (BrdU/NeuN co-localization) was much shorter - 38 days (42 days post-injury survival minus 4 days post-injury start of BrdU administration). The time window of 38 days after BrdU injection in the present study should have been sufficient to detect at least DCX positive immature neuroblasts in the MC or in the primary neurogenic sites – the SVZ. Alternatively, the quantity of SVZ neurogenesis destined to replace cortical neurons might be too low to be identifiable in the current experimental setting.

The failure to demonstrate neurogenesis in the adult spinal cord confirms numerous previous reports [22]–[29]. Cell proliferation and reactive gliogenesis have been shown in regions close to the lesion site [24], [35], [53]. As sources of glial renewal cells around the central canal, NG2 positive glial progenitor cells in the white and grey matter and mature GFAP expressing astrocytes have been identified [54]. The observed glial cell replacement is paralleled by ongoing glial cell death in spinal cord regions, not only adjacent but also remote from the injury site [20], [55], [56]. In monkeys, sites of oligodendrogliogenesis correlate with demyelinated areas suggesting that replaced oligodendroglia contribute to remyelination and possibly functional recovery in incomplete SCI [57]. In the present study, glial replacement (astroglia, oligodendroglia, microglia) in the cervical spinal cord exceeded gliogenesis compared to uninjured control animals. It is conceivable that remote areas of demyelination become spontaneously remyelinated via endogenous oligodendroglial replacement. Whether remote demyelination or potential remyelination by replaced oligodendroglia influence the functional outcome, cannot be determined at this point. Astroglial renewal immediately at the spinal cord lesion site is attributed to reactive astrogliosis, which seals off the injury site from the surrounding spinal cord. The relevance of astroglial turnover observed at remote spinal cord levels has yet to be defined. Wallerian degeneration of ascending sensory projections at cervical spinal cord level represents a potential trigger, which might explain enhanced microglial cell recruitment following thoracic SCI.

### Conclusion

The true functional relevance of the observed glial replacement in spinal regions distant from the injury site is unknown. However, considering the robust spontaneous recovery in contrast to the moderate cell replacement activities in the cervical spinal cord, it is unlikely that endogenous cell replacement contributes to spontaneous functional improvement in incomplete SCI.
